# Immunologic Rejection of Transplanted Retinal Pigmented Epithelium: Mechanisms and Strategies for Prevention

**DOI:** 10.3389/fimmu.2021.621007

**Published:** 2021-05-12

**Authors:** Carson C. Petrash, Alan G. Palestine, M. Valeria Canto-Soler

**Affiliations:** ^1^ CellSight Ocular Stem Cell and Regeneration Research Program, Department of Ophthalmology, Sue Anschutz-Rodgers Eye Center, University of Colorado School of Medicine, Aurora, CO, United States; ^2^ Department of Ophthalmology, University of Colorado Anschutz Medical Campus, Aurora, CO, United States; ^3^ Charles C. Gates Center for Regenerative Medicine, University of Colorado School of Medicine, Anschutz Medical Campus, Aurora, CO, United States

**Keywords:** retinal pigment epithelium (RPE), induced pluripotent stem cells (IPSC), age-related macular degeneration (AMD), transplant rejection, immune rejection, transplant acceptance, retinitis pigmentosa, immunosuppression

## Abstract

Replacement of dysfunctional retinal pigmented epithelium (RPE) with grafts derived from stem cells has the potential to improve vision for patients with retinal disorders. In fact, the potential is such that a great number of groups are attempting to realize this therapy through individual strategies with a variety of stem cell products, hosts, immunomodulatory regimen, and techniques to assess the success of their design. Comparing the findings of different investigators is complicated by a number of factors. The immune response varies greatly between xenogeneic and allogeneic transplantation. A unique immunologic environment is created in the subretinal space, the target of RPE grafts. Both functional assessment and imaging techniques used to evaluate transplants are susceptible to erroneous conclusions. Lastly, the pharmacologic regimens used in RPE transplant trials are as numerous and variable as the trials themselves, making it difficult to determine useful results. This review will discuss the causes of these complicating factors, digest the strategies and results from clinical and preclinical studies, and suggest places for improvement in the design of future transplants and investigations.

## Introduction

Retinal diseases with degeneration or dystrophy of photoreceptors are visually devastating and there are no current therapies to regenerate retinal tissue. In age-related macular degeneration (AMD) and some forms of inherited retinal diseases (IRDs) such as Best Disease and MERTK-associated Retinitis Pigmentosa (RP), the primary dysfunction affects the retinal pigmented epithelium (RPE) (1–3). Adjacent to the neuroretina, the RPE is responsible for supporting the metabolism of light-sensing photoreceptors, regenerating 11-cis-retinol for the visual cycle, forming the outer blood-retinal barrier, and reducing light scatter (4). Degeneration of RPE leads to secondary loss of photoreceptors and subsequent permanent loss of vision (2, 3). The latest research seeks to treat these degenerative conditions by transplanting healthy RPE at early stages of disease.

Sources of RPE for transplantation have evolved through the years. Pioneering investigations studied primary cultures of RPE (5); however, more recent investigations have used RPE derived from stem cells due to the capacity for unlimited self-renewal and greater possibility of selecting for desired characteristics (6). Stem cell sources of RPE include RPE stem cells (RPESC) found in the adult native tissue (7), embryonic stem cells (ESCs), and induced pluripotent stem cells (iPSCs) (6). This review will primarily focus on studies involving transplantation of RPE derived from ESCs and particularly iPSCs as they have the greatest potential as graft tissue. Autologous transplant iPSC-RPE is currently under clinical trial [(8, 9), and ClinicalTrials.gov # NCT04339764]. Due to genetic variability, instability, and potential tumorigenicity of iPSCs, validation of an autologous cell line is a long and costly process that will be challenging to scale for treatment of the millions of potential recipients (10). Alternative approaches and preclinical investigations involve xenogeneic or allogeneic sources and carry heightened risk of immunologic rejection (11).

The eye as a whole represents a unique immunologic environment. The “immune privilege” of the eye is not what it was once hoped; nonetheless, features of the subretinal space (SRS) create a relatively safe target for transplantation of iPSCs (12). In addition, both stem cells and RPE are attributed with anti-inflammatory properties that enhance compatibility (13, 14). Nevertheless, stem cell derived RPE is susceptible to immune rejection from days to months following transplantation (15–17). Effector mechanisms of acute (i.e., after days) and chronic (weeks to months) are interrelated but distinct (18). For this broad problem of immune rejection, a one-fits-all solution is unlikely and multiple strategies for prevention are necessary.

The mere determination of rejection of an RPE graft is controversial. Subretinal biopsy for histologic confirmation is implausible, and the small volume of grafted tissue may be insufficient to produce systemic manifestations. Researchers instead rely on longitudinal functional tests and imaging techniques. Recent investigations are revealing that these surrogate markers are susceptible to confounding results (15, 19–21). No prior authors have comprehensively reviewed the common signs or alternative phenomena that can masquerade as immunologic rejection or lack thereof. Beyond determining rejection of RPE transplants, very little is known regarding how it may be prevented. To the best of our knowledge this is the first review to specifically compare immunomodulatory regimen and results and attempt to identify trends. Furthermore, new directions will be discussed, and specific strategies proposed to prevent rejection of RPE transplants in future trials.

## Section I: Transplant Immunology

Rejection of solid transplants can result from any of a series of actions of the innate, nonspecific and the acquired, antigen-specific immune systems (22). Innate immunity includes cellular elements: macrophages, neutrophils, natural killer cells, and resident lymphocytes which can cause acute rejection within the first week (16). These cells also express pattern-recognition receptors (PRRs) such as toll-like receptors (TRLs) which allow them to recognize damage-associated molecular patterns (DAMPs) released as a result of tissue damage, including graft surgery or early rejection (23). Binding of the PRRs to DAMPs activates immune cells and potentiates inflammation, including expression of antigen presenting cells (APCs) and CD4 T-cells. T-cells in particular orchestrate the acquired cellular and humoral immune cascade to reinforce the innate response, generating an expansion in both CD8 “cytotoxic” T-cells and antibody-producing B-cells. These responses by lymphocytes cause chronic rejection of transplants beyond the first week (18).

Early work in solid organ transplants demonstrated that T-cells are necessary and sufficient for allograft rejection (24–26). Subsequently, immunosuppressive regimen were developed that target T-cell activation at three steps: signal 1) binding by APCs, signal 2) costimulatory molecules and ligands, or signal 3) the trigger for cell proliferation (27). Most common agents include tacrolimus and cyclosporine: calcineurin inhibitors that prevent signal 1. Antimetabolites such as mycophenolate mofetil (MMF) target signal 3. Likewise for RPE transplant studies, the most frequently used drug is tacrolimus. Glucocorticoids including prednisone, dexamethasone, and triamcinolone are prescribed as well, which have broad effects on T-cells by reducing expression of MHC II (signal 1), increasing percentage of regulatory T-cells, and inducing T-cell apoptosis (signal 3) (28–30). Despite these measures to modulate acquired immunity, RPE grafts routinely exhibit signs of rejection weeks to months after transplantation, and histology recovered from these eyes confirms a predominance of lymphocytic infiltrate (15, 17, 20, 31–35). Evidently there is room for improvement in understanding the immunologic response to subretinal RPE grafts.

In solid organ transplants, infiltration of mononuclear phagocytes (MNP) is a prominent feature of rejection (36). MNPs include bone marrow derived macrophages (BMM) and tissue resident macrophages (TRMs). Greater number of BMM correlates with worse clinical outcomes (37, 38) and depletion of BMM improves allograft function (39). BMM represent the majority of macrophages in rejected solid organ transplants (40). Similarly, MNPs form a large component of the cellular infiltrate of rejected RPE grafts (15–17, 20, 32, 33, 35). This is especially true when rejection of RPE occurs within the first week, prior to the development of acquired immunity (15, 16). Transplant immunologists have increasingly looked at MNPs to gain a better understanding of allograft rejection (26).

Compared to BMM which infiltrate inflamed tissue, TRM are critical to healthy tissue homeostasis. Microglia are the TRM of the central nervous system and retina and perform functions including phagocytosis of waste and regulation of vascular and neural growth (41, 42). In non-diseased retina, microglia are located in the inner and outer plexiform layers but are absent in the SRS (4). In IRD (43) and light-induced degeneration models (44), microglia were the dominant MNP lineage in the SRS, while BMM were largely limited to the neuroparenchyma. We speculate that this may underscore the relative importance of microglia in the immune response to transplanted RPE compared to other solid organ transplants given that recipients may have a pre-existing or predisposition to microglial infiltrate. Furthermore, the avascularity of the graft and SRS represents an additional barrier to BMM infiltration of RPE grafts compared to solid organ transplants. Nonetheless, both microglia and BMM are likely to play important roles given their prevalence in inflammatory infiltrate around rejected RPE (15–17, 20, 32, 33, 35).

**Figure 1:**
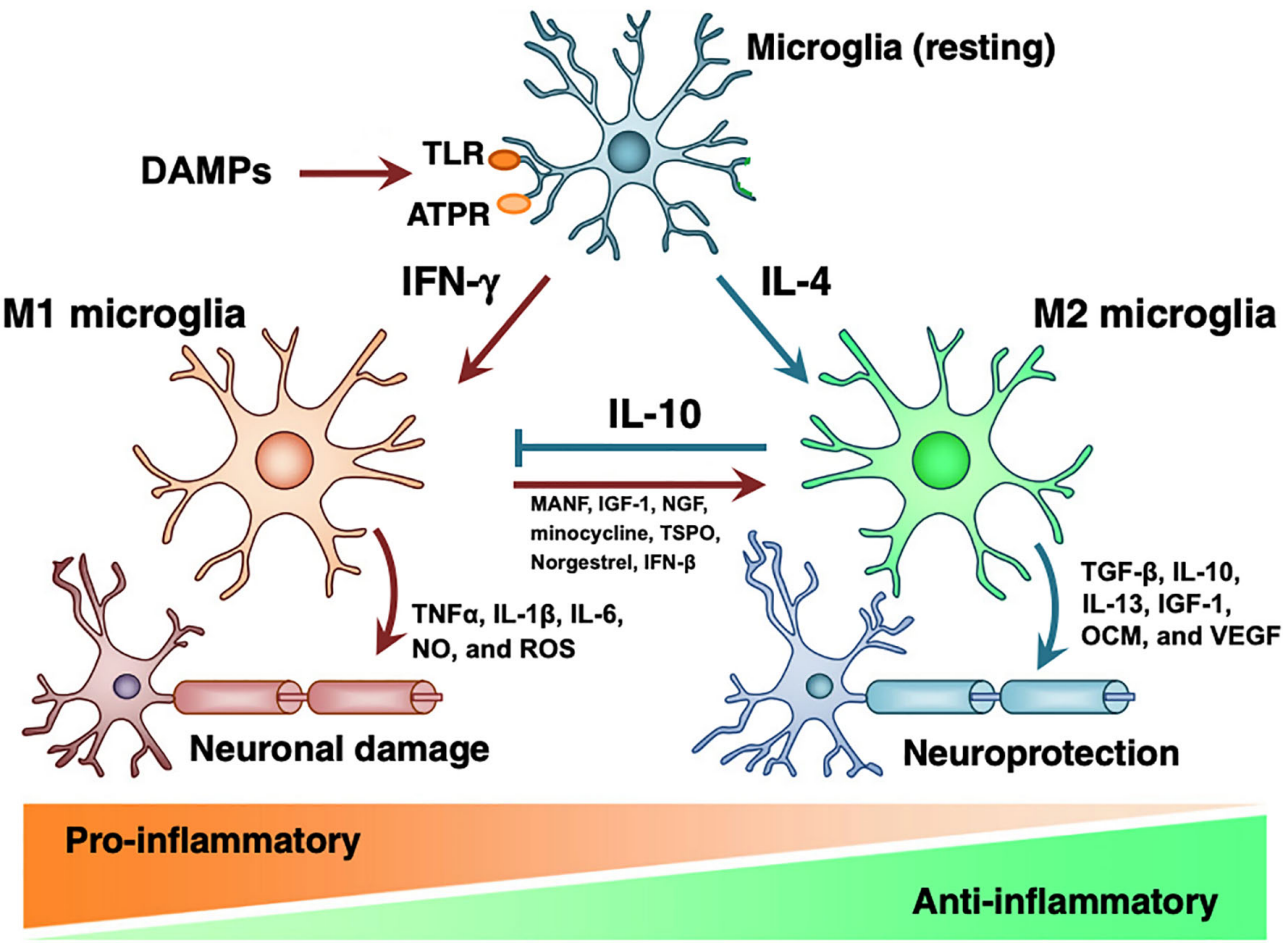
Microglial M1 and M2 phenotypes. DAMPs act on PRRs on resting microglia to induce formation of M1 or M2 microglia. M1 microglia induce pro-inflammatory state and tissue damage by releasing TNFα, IL-1β, IL-6, nitric oxide, and ROS. M2 microglia have tissue-protective effects *via* TGF-β, IL-10, IL-13, IGF-1, OCM, and VEGF. While IL-10 inhibits M1 microglia, introduction of MANF, IGF-1, minocycline, TSPO, norgestrel, and IFN-βB induce transition to M2 phenotype. Modified from Nakagawa 2015 with notes from Kramer 2019, Elsevier license 4858840650798 (41, 47).

As the resident immune cells the graft site, TRM may direct the initial response to transplanted grafts (16, 26, 45, 46). Depicted in **Figure 1**, *in vitro* studies have demonstrated that DAMPs released as a result of transplantation surgery bind to TLRs and cause IFNγ driven proliferation of M1 microglia, which release TNFα, IL-1β, IL-6, nitric oxide, and reactive oxygen species to propagate inflammation and neural damage (48, 49). If the inciting trauma resolves, IL-4 induces proliferation of the M2 phenotype of microglia. M2 microglia produce anti-inflammatory TGFβ, IL-10, IL-13, Ccl2 (50–53), neurogenic oncomodulin (OCM), insulin-like growth factor (IGF)-1, and vascular endothelial growth factor (VEGF), returning the retina to healthy state (54, 55). However, if the original injury persists, the inflammatory M1 phenotype causes progressive tissue damage (41). To corroborate this *in vitro* evidence, *in vivo *studies have indicated that allograft transplantation without immune suppression is associated with persistent M1-type BMM, while macrophages rapidly convert to M2-phenotype with adequate immune suppression (26, 46, 56). In summary, we hypothesize that surgical trauma causes proliferation of destructive M1 microglia that contribute to acute rejection, and dysregulation of the M1 phenotype leads to macrophage infiltrate surrounding chronically rejected RPE grafts.

Due to anti-inflammatory and neuroprotective properties of M2 phenotype microglia, we speculate that supporting the transition from M1 to M2 may be an effective strategy to reduce immune rejection and improve retinal function. As shown in **Figure 1**, there are many potential therapies that stimulate the expression of M2 microglia, including mesencephalic astrocyte-derived neurotrophic factor (MANF) (41, 57), growth factors IGF-1 and NGF (58, 59), minocycline (60, 61), translocator protein (TSPO) (62), norgestrel (63, 64), and IFN-β (65). With the exception of minocycline, none of these therapies have been adopted in RPE transplant studies (66). Glucocorticoids have been included in RPE transplant trials and are known to reduce production of inflammatory cytokines IL-1 and TNFα and inhibit macrophage phagocytic function; however, the relative role of these pro-M2 functions compared to the effects of macrophages on T-cells and acquired immunity is undetermined (67–69). Therefore, specifically targeting microglial phenotype represents largely untested waters with potential benefits for both reducing immunologic rejection and improving the health of transplanted neurons.

Clinical and preclinical investigations to date have focused on inhibition of acquired immunity to prevent rejection; however, given the 1) variable chronicity of rejection, 2) prevalence of microglial/macrophage infiltrate on histology of rejected transplants, and 3) established immune physiology of the retina, we propose that is important to consider specifically targeting M2 macrophage phenotype. This strategy should be investigated first in pre-clinical studies. The primary effectors of rejection may depend on the level of transplantation. Due to the greater phylogenetic difference, xenografts may be more readily recognized by innate immune system and susceptible to acute rejection. Allografts that avoid innate recognition may have to evade the acquired immune system or else succumb to chronic rejection. Differences in strain (e.g., Yucatan pig versus P23H minipig) or immunophenotypes (e.g., MHC antigens) may also determine the chronicity and effectors of rejection. However, given that macrophages and T-cells secrete cytokines that affect one another’s phenotype, i.e. M1 versus M2 macrophage or Th_1_/Th_2_ versus regulatory T-cells, we hypothesize that both T-cell inhibitors and M2 promoters offer immunologic benefits throughout the postsurgical period.

Two further strategies of transplant immunology warrant a mention. First, grafts composed of tissue constructs have demonstrated greater success than cellular suspensions, as demonstrated by the success of pancreas compared to islet cell transplants (70). Furthermore, there is evidence that hESC-RPE transplants may survive longer as a monolayer (71) and many current trials have adopted this strategy (66, 72, 73) and [ClinicalTrials.gov # NCT04339764]. Nonetheless, this is still up for debate (74) and even recent trials have delivered RPE cell suspensions (15, 35). Drawing conclusions regarding the ideal form of delivery is also complicated by the variable scaffolds on which RPE monolayers are implanted, and the possibility that they create a barrier to diffusion of metabolites from the choroidal vasculature. Secondly, long-term survival of solid organ transplants can be achieved without immune suppression through the infusion of hematopoietic stem cells (HSCs) at the time of transplantation. Transfused HSCs engraft within the thymus and bone marrow of the recipient and cause production of host blood T- and B-cells that are tolerant of graft antigens. While this effect has been demonstrated in both animal and human recipients of solid organs, its potential has not been tested with respect to RPE grafts (75).

## Section II: Sub-Retinal Immune Response and Privilege

The eye is unique among tissues in that its function is dependent on the optical clarity of its media: cornea, aqueous, lens, and vitreous. To prevent excessive inflammatory changes, the eye has physiologic systems that are distinct from the systemic immune system. In 1905 these mechanisms allowed the first successful corneal transplant before the advent of immunomodulatory medications, leading to the concept of “immunologic privilege”. However, privilege of the SRS has been over-stated in the past: even allografts transplanted with immunomodulation are susceptible to rejection. Nonetheless, it is worthwhile to consider the mechanisms of immune privilege in order to design interventions. The physiology of immune privilege is extensive and only few features will be defined here, organized into three groups as detailed by Caspi et al., 2006: separation, local inhibition, and systemic regulation (76).

### Immune Separation

Separation refers to the physical blood-retinal barrier (BRB) that prevents systemic immune cells and large molecules from passing into ocular tissues. The barrier is primarily created by intercellular tight junctions. An outer barrier is formed between cells of the retinal pigmented epithelium (RPE), while an inner barrier is formed between endothelial cells of the inner retinal vasculature. In addition to trauma, the BRB can be weakened by inflammation and neovascularization (77).

Surgical approaches for graft placement are transscleral or transretinal. While the transscleral approach violates the outer BRB, transretinal placement requires vitrectomy, retinotomy, and endolaser: all procedures associated with production of DAMPs and inflammation, thus stimulating proliferation of M1 microglia and subsequent tissue damage (78). Therefore, it is yet undetermined whether a transscleral or transretinal approach is immunologically preferable. However, subjects with retinal disease may be predisposed to rejection due to inflammation associated with their primary disorder (e.g., AMD or RP) or prior intraocular surgeries (79).

Based on what we know regarding immune separation, measures may be hypothesized to minimize rejection in the SRS. First, ideal study subjects should have no intraocular inflammation or neovascularization (77). Retinal diseases with degenerative or dystrophic RPE may increase risk as well. Secondly, the implant should be placed far from the retinotomy or Bruch’s membranotomy to minimize M1 microglia in the location of the graft (41). Lastly, it is worth emphasizing that tissue trauma should be minimized (41, 77).

### Local Inhibition

Disruption of the BRB leads to influx of reactive cells from the systemic circulation, and thus local inhibition of the invading cells and inflammatory mediators is necessary to prevent the escalation of inflammation. Native RPE has a primary role in this anti-inflammatory “effector blockade” (76). Cytokines produced by RPE include TGFβ, IL-11, and IFNβ (80). In addition to enhancing the M2 microglial phenotype, TGFβ is responsible for inhibiting the action of inflammatory T-cells and promoting development of regulatory T-cells (81, 82). IL-11 has cytoprotective and anti-inflammatory functions (83). IFNβ inhibits expression of cellular adhesion molecules and chemokines sICAM-1 and CXCL9, which attract T-cells and NK cells to sites of inflammation (80). With regards to membrane-bound receptors, Fas ligand (FasL) and PDL1 are highly-expressed on RPE and responsible for inducing apoptosis of invading T-cells or converting them to regulatory T-cells (81, 84). CD46 on RPE prevents the activation of the complement cascade (76).

Paradoxically, RPE also has pro-inflammatory behavior. The most significant of these mechanisms is its role as an antigen presenting cell (APC) with MHC-II, a feature shared only by specific immune cells (85). This introduces the paradox that it may be beneficial to transplant atop RPE atrophy, despite the lack of an outer BRB, due to potentially reduced antigen presentation. No study has addressed this question. RPE has a vital role in the local control of inflammation in the SRS and targeting expression of MHC-II, TGFβ, or any of the local immune inhibitory factors of RPE may affect survival of grafted RPE (33).

### Systemic Regulation

In states of trauma or inflammation, immune cells are able to pass the physical barriers and evade the local inhibitory mechanisms to access the SRS and subsequently re-enter systemic circulation. In the non-privileged immune response, APCs take antigens from the site of inflammation to local lymph nodes or the spleen where they activate delayed-hypersensitivity (DH) T-lymphocytes, potentiating the inflammatory response. The eye has mechanisms to regulate this reaction as well. Similar to the well-studied anterior chamber-associated immune deviation (ACAID), CD11b^+^ microglia in the retina induce the development of regulatory T-cells in the spleen, which dampens the response of DH T-cells (86, 87). Despite years of study, ACAID has never been utilized for therapeutic effect. The physiology of ACAID suggests that it may be possible to stimulate regulatory T-cells and inhibit rejection by priming the host with graft antigens, similar to how HSCs engraft to induce tolerance to solid organ transplants. This remains an area for future study.

The relative role of immune separation, local inhibition, and systemic regulation in preventing rejection of transplanted RPE in the SRS is undetermined. Furthermore, the strategies that we propose for preserving immune separation are also unproven and alone insufficient to prevent rejection. However, understanding the immune mechanisms in the SRS will assist researchers in designing investigations and strategies, and our recommendations are compatible with further interventions that, together, may improve success of future trials.

## Section III: Evaluating Rejection and Lack of Rejection

Most organ transplants can be considered to not have been rejected by the host if the graft is performing its physiologic function: for example, a kidney producing urine, liver metabolizing toxins, or a lung exchanging gases. This is more difficult to define in the retina. For one, animal subjects are not able to comply with standard tests for visual function such as acuity or field tests. While human patients are able to complete these tests, results may be confounded by some degree of photoreceptor functional rescue induced by growth factors released as a byproduct of retinal detachment or other elements of the surgical procedure (88, 89). Therefore, improved acuity, fields, or even electrophysiologic response may be due to surgically induced cellular repair processes rather than function of transplanted cells. Secondly, there are no clear systemic parameters that indicate rejection of a retinal transplant. Elevated inflammatory cells in the blood can indicate rejection of a solid organ; however, stem cell RPE grafts may undergo a slow functional deterioration or rejection without manifest signs of inflammatory reaction (15, 21), perhaps due to the anti-inflammatory properties of the RPE graft or immune privilege mechanisms of the SRS (19).

**Figure 2:**
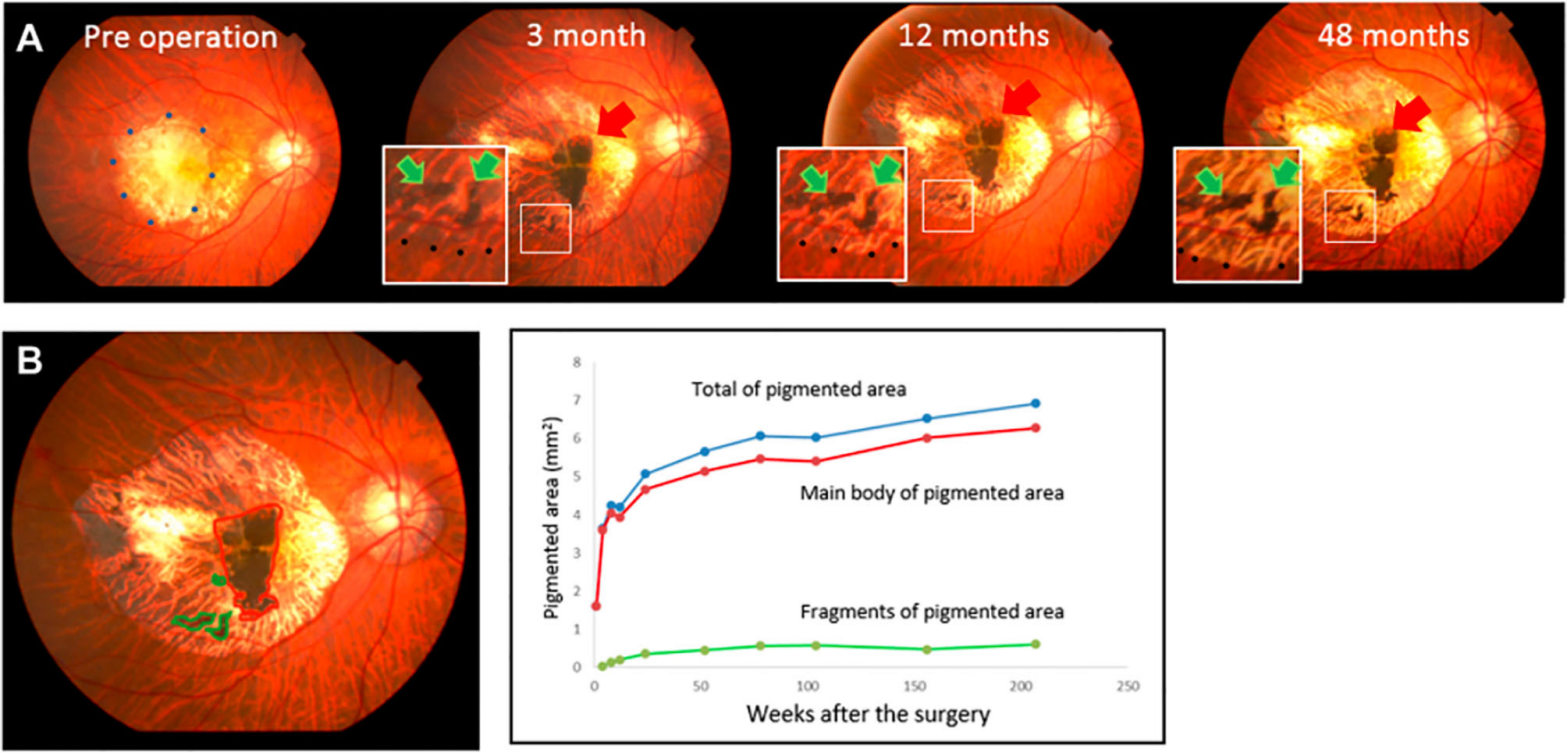
Photographic fundus images over four years following transplant of sheet of autologous iPSC-RPE. **(A)** Red arrows point to the main graft while green arrows indicate islands of graft cells. **(B)** The graft area, identified by the presence of pigmentation, was calculated using ImageJ. Areas of the main graft and islands of grafted cells were plotted over 4 years. Expansion of pigmented area is exhibited in relatively homogenous distribution without clumping or signs of inflammation. Reprinted from Takagi 2019, license number 4867110135570.

**Figure 3:**
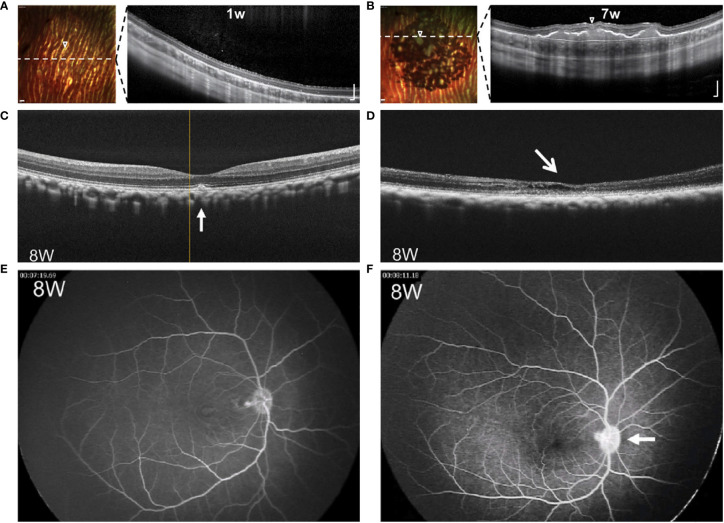
Comparative images of RPE grafts with and without rejection. **(A, B)** Multicolor-confocal scanning laser ophthalmoscopy and corresponding OCT following subretinal injection of RPE cell suspension. Homogenous monolayer leads to clumping of pigment over 1-7 weeks. Dashed line indicates plane of corresponding OCT, where subretinal mass is seen along with choroidal thickening under the graft site. Modified from Petrus-Reurer 2020 (35). **(C, D)** OCT eight weeks following transplant of RPE suspension. Compared to non-rejected transplant **(C)**, retina with rejected graft **(D)** shows atrophy of the neuroretina with intraretinal and subretinal fluid. **(E, F)** FA eight weeks following transplant of RPE suspension. Compared to non-rejected transplant **(E)**, retina with the rejected transplant **(F)** shows leakage of the optic disc. Modified from Sugita 2016 (33).

In contrast to these challenges, retinal transplants have advantage of visibility with ophthalmoscopy. Studies from autologous transplants are perhaps the best resource to demonstrate the appearance of RPE grafts in the absence of rejection. As shown in **Figure 2**, a flat, expanding, pigmented monolayer is observed after transplantation of autologous iPSC-RPE (8, 9). In allogeneic clinical trials, Mehat 2018 found dose-dependent hyperpigmentation at the site of transplantation, and this study along with Schwartz 2016 observed a predilection for hyperpigmentation in areas of prior retinal atrophy, possibly representing migration of donor cells (21, 90, 91). While it may be true that successfully transplanted RPE would have this morphology, pigmented cells can also represent macrophages that have phagocytosed rejected or apoptotic RPE or host RPE that proliferates in response to the transplant surgery (92, 93). Mehat 2018 noted that their findings would also be consistent with subretinal spread of released pigment (21, 90). On the other hand, the lack of obvious pigmentation does not mean that transplanted RPE has been rejected, as RPE may have variable pigmentation (21, 94, 95). Schwartz 2016 found no correlation between pigmentation and changes in visual function (21). Nonetheless, serial fundus photographs may manifest changes when RPE is rejected (20). Clumping of pigment or pigment-laden cells has been reported and suggested as a sign of rejection, as seen in **Figure 3** (32, 35, 73). The appearance of the grafted tissue is a valuable parameter when determining the immunologic success of RPE transplantation, but it must be considered that appropriate morphology and pigmentation is not sufficient.

Along with direct visualization techniques, the retina allows imaging with techniques such as optical coherence tomography (OCT), fluorescein angiogram (FA), and fundus autofluorescence (AF). OCT enables cross-sectional visualization of neuroretina layers, transplanted tissues, and changes such as edema or retinal detachment. Images demonstrating both normal morphology and representative changes associated with rejection are seen in **Figure 3** and compiled in **Table 1**. Features that suggest lack of rejection include: 1) the presence of transplanted cells as a homogenous monolayer or hyperreflective outer retinal band (21, 35, 66, 72, 73), and 2) improved retinal morphology including formation of an external limiting membrane and outer nuclear layer thickness of greater than 20 um (34, 72). However, the presence of a hyper-reflective outer retinal band may persist following cellular rejection (15), and improved morphology may also result from surgically-induced changes (88). On the other hand, features that indicate rejection include: 1) signs of inflammation such as cystoid macular edema (CME), choroidal thickening, hazy vitreous, 2) deposition of subretinal material, 3) neuroretina atrophy, and 4) disintegration or clumping of transplanted cells (34, 35, 66, 72, 73). Nevertheless, it must be considered that rejection may occur without manifest signs of inflammation (15, 19), and inflammation or retinal atrophy can occur due to primary ophthalmic disease or the surgery alone in the absence of rejection.

**Table 1:** Comparison of visual signs of rejected versus non-rejected transplanted RPE.

	Rejection	No Rejection
Fundoscopy	Clumping of pigment Optic nerve head hyperemia	Expanding, flat, pigmented layer
OCT	Inflammation: CME, choroidal thickening, vitreous haze Subretinal deposits Neuroretina atrophy Disintegration or clumping of transplant material	Hyperreflective monolayer Healthy neuroretina morphology: -Intact external limiting membrane -Outer nuclear layer > 20 um
FA	Optic nerve head leakage Leakage around graft	Absence of leakage around graft or optic nerve head
AF	Uneven autofluorescence Loss of autofluorescence over time	Double thickness of autofluorescence (transplanted over host RPE)

Fluorescein angiography (FA) is used to reveal abnormal vasculature or changes in RPE. When used to assess RPE grafts, signs that indicate rejection are leakage of dye around grafts, CME, and optic disc edema, as seen in **Figure 3** (33). Leakage around grafts can be associated with surgical trauma to vasculature or Bruch’s membrane or to the primary disease of the host: for example, central serous chorioretinopathy, which would increase likelihood of subsequent rejection. Again, since rejection may occur in the absence of inflammation (15, 19), destruction of the graft may occur without leakage or CME. Del Priore 2003 demonstrated no difference in serial FAs between immunocompetent and immunomodulated hosts despite the greater speed of rejection in the former (20).

Fundus autofluorescence (AF) is often used in RPE transplant studies to visualize lipofuscin and RPE pigments. Da Cruz 2018 reported a double-thickness of autofluorescence where host RPE overlapped native RPE, followed by uneven autofluorescence attributed to phagocytosis of RPE cells by immune cells (73). While AF cannot differentiate between host and transplanted RPE, a similar technique, green fluorescent protein (GFP), can make this distinction and is frequently applied to basic research. GFP is used to determine the survival percentage of transplanted RPE or follow their rejection by the loss of fluorescence over time (15, 31, 32, 66). Unfortunately, *in vivo* immunogenicity of GFP may cause cytotoxicity over time, meaning that transplanted GFP-positive RPE would be more susceptible to rejection (96).

Two recent studies proposed an alternative to visual techniques by correlating systemic donor specific antibody (DSA) with rejection of transplanted RPE (17, 35). The importance of DSA is well known in other areas of transplant medicine (97–99), and as described in Section I of this review, may have even more importance in rejection of subretinal RPE grafts. Sugita 2017 demonstrated reactivity of peripheral blood mononuclear cells (PBMCs, i.e. lymphocytes and monocytes) correlated with rejection of grafted RPE after eight weeks (17). Another serology with undetermined potential in RPE transplants: cell free DNA has been used to follow rejection of renal transplants (100). In the field of RPE transplantation where defining rejection or compatibility depends substantially on qualitative assessments, these objective measures should be considered for further applications.

There is no way to conclusively determine rejection or compatibility of transplanted RPE without histologic analysis, which can realistically only offer an endpoint. Functional tests are confounded by rescue of native photoreceptors. Fundoscopy is essential, but the presence of pigmentation has various causes. OCT, FA, and AF all provide important diagnostic clues, but rejection can occur in the absence of post-operative changes and inflammation can be due to ophthalmic disease or surgery. Furthermore, with functional tests and imaging it is not possible to differentiate immunologic rejection from cellular degeneration due to non-immunologic mechanisms. Serologic analysis of DSA, PBMCs, and GFP may offer objective evidence of rejection or survival but are infrequently used. For the time being, we must closely follow the trends that may yield more conclusive evidence of transplant function and survival in the host retina.

## Section IV: Preventing Rejection of RPE Grafts

The literature demonstrates three strategies to prevent immune rejection of RPE grafts: 1) immunodeficient host models, 2) pharmacologic immune modulation, and 3) reducing the immunogenicity of the graft. Each of these strategies has particular applications. Immunodeficient hosts are ideal for demonstrating functional potential of transplants and tumorigenicity of stem cell transplants in the absence of immune rejection. Without additional immune modulation, xenogeneic ESC-derived RPE survived for at least 240 days in the NIH III mouse, deficient in T-, B-, and NK cells (95), while ESC- and iPSC-derived RPE grafts have survived up to 12 months in the athymic nude rat (66, 71, 101). No studies of RPE-grafts have been performed in immunodeficient large mammals such as non-human primates or pigs, possibly related to a deficiency of these models or poor translation to clinical medicine.

Except in the cases of autologous or MHC-matched grafts, every study that has demonstrated evidence of temporary survival of RPE transplants in immunocompetent hosts has applied pharmacologic immune modulation. With respect to xenogeneic studies, Del Priore 2003 demonstrated “triple systemic” therapy with prednisone, cyclosporine, and azathioprine (anti-inflammatory antibiotic) to increase the survival of grafted RPE at four weeks (20). Similar results were obtained when the host was exposed to local cyclosporine alone, as a weekly intravitreal injection or slow-release capsule (31). Graft survival was prolonged to 100 days when cyclosporine was administered from pre-op day two until day 100 along with dexamethasone for two weeks (94). However, grafts survived less than four weeks in a similar study with a higher concentration and duration of dexamethasone (102), which may suggest the greater role of systemic cyclosporine compared to systemic steroids in preventing rejection. Local administration of steroid demonstrated a benefit with intravitreal triamcinolone administered at the end of the surgery and improved survival after four weeks compared to hosts that received daily intramuscular dexamethasone (102). This effect was repeated by a subsequent study, with the additional finding that intravitreal tacrolimus improved the morphology of RPE on OCT (34). Typically used as antibiotics, doxycycline and minocycline have been used for suppression of microglia of the innate immune system (61, 103) and were associated with 70% survival of grafted RPE after 10 weeks when administered with prednisone, tacrolimus, and sirolimus. Given coadministration with steroid and T-cell inhibitors, it is not possible to determine their specific effects from these studies.

Regarding allogeneic transplants, preclinical trials were performed with iPSC-RPE in pigs and macaques: the former with no immune modulation and the latter with subconjunctival and topical dexamethasone. Both investigations demonstrated histologic evidence of robust rejection in the period between postoperative day four and three weeks, reinforcing the need for appropriate immunomodulatory regimen in allogeneic iPSC trials (15, 32). The best evidence regarding local steroids comes from Sugita 2017, where a regimen of intravitreal triamcinolone at the time of surgery and sub-tenon triamcinolone at four weeks post-op prevented development of DSA, reactive PBMCs, and inflammatory infiltrate at six months compared to a control monkey without immunomodulation. In fact, these results were similar to an MHC-matched subject (17).

The remainder of allogeneic trials in the literature pertain to humans and subsequently lack histologic confirmation of rejection or compatibility. Four clinical trials administered tacrolimus with or without mycophenolate mofetil (MMF), an antimetabolite with favorable side effect profile (21, 72, 90, 93). Dosing of both tacrolimus and MMF started one week prior to surgery and continued for up to 13 weeks before withdrawal; however, MMF takes several months to become fully effective, so this may represent underutilization of the effect of this drug. A fourth clinical trial used perioperative prednisone with an intraocular steroid implant (73). If proven to prevent rejection in further studies, this could be an option for geriatric patients in whom systemic immune modulation may increase risk of complicating infection. All of these clinical trials demonstrated evidence of survival of the transplant past the cessation of pharmacologic immunomodulation but lack histologic confirmation.

Reducing the immunogenicity of an RPE graft is a primary objective of both preclinical and clinical trials. In immunologic terms, the ideal graft is autologous, or composed of tissue from the host. iPSC technology is currently used to create autologous RPE grafts derived from adult cells for clinical trials in Japan and the US [(8, 9, 104) and ClinicalTrials.gov # NCT04339764]. When transplanted into a human host without immunomodulation, an autologous iPSC-RPE graft survived for over one year and was associated with preserved neuroretina structure (8, 9). Despite these apparent successes, there are many barriers to widespread clinical application of autologous iPSC lines. High genetic variability and instability increases the risk of immunogenicity and tumorigenic potential in iPSCs (10). Properly verifying a new iPSC cell line may require 12-15 months and approximately $800,000 (105, 106). Perhaps a more viable option in the future, autologous-iPSC lines are not currently available for the majority of researchers or patients.

In lieu of autologous transplants, many authors have proposed banking of high-quality iPSC-RPE lines that can be matched for allogeneic transplants to patients (10). Banks of 10, 55, 80, and 150 donor iPSCs could match over 40% of genotypes in Korea, 80% in Japan, 50% in California, and 93% in the UK, respectively (41, 107–110). As evidence for this approach, when MHC-matched macaque-iPSC-RPE was transplanted in the SRS of allogeneic hosts, grafts survived without immune suppressants for 6 months and were associated with no development of DSA, reactive PBMCs, or infiltration of inflammatory cells compared to MHC-mismatched controls (33). A potential alternative studied by Petrus-Reurer 2020 is MHC-II knock-out hESC-RPE, which delayed the infiltration of inflammatory cells and production of DSA compared to controls with wild-type hESC-RPE (35). Knockout of β2-microglobulin and MHC-I were also studied, and reduced activation of NK cells compared to controls (111, 112). Notably, expression of MHC-II by RPE is also reduced by culture under xeno-free conditions, emphasizing the use of these methods to decrease rejection as well as comply with clinical requirements (34, 35). Significantly, donor RPE cannot present antigen to human hosts; however, modifying donor cells to be less immunogenic is compatible with the design of iPSC-RPE cell banks to increase the number of patients who may match to a limited number of cell lines.

In addition to the cellular components of a graft, the immunogenicity of the transplant vehicle is an important consideration. Biodegradable scaffolds offer the potential benefit of reducing a chronic foreign body reaction but have potential disadvantages as well. Poly(lactic-co-glycolic acid) (PLGA) degrades into components lactic and glycolic acid, which are potentially toxic, but did not produce signs of inflammation on OCT 5 weeks after delivery (66). Fibrin is biochemically inert, but the mechanically suitable fibrin scaffold is approximately 200 µm thick, raising concerns regarding the ability of nutrients and waste to diffuse to and from the choriocapillaris (113). Nonetheless, when implanted in the SRS of pigs, the overlying neuroretina appeared to be healthy when the scaffold degraded after eight weeks.

Non degradable scaffolds include parylene, polyester, and polyethylene terephthalate (PET). Parylene increased survival of RPE transplants relative to cells delivered as a suspension (72, 114). Implantation of an acellular polyester membrane into the SRS was associated with a foreign body reaction with chronic inflammatory infiltrate; however, the presence of RPE on the membrane reduced this response (73). Following implantation of a PET acellular membrane, microscopy demonstrated an additional cellular layer between the host RPE and the PET membrane, attributed to reactive migration of host RPE or infiltration of macrophages (34). Immunologic consequences of these delivery materials are likely to be relatively more significant as advancements are made in autologous or universal RPE cell banks.

## Discussion

For years limited to basic science research, stem cells therapy for patients with retinal disease is currently in clinical trials. However, while treatments are seemingly close at hand, the mere survival of grafts is still a controversial determination. To reach millions of patients with retinal degenerations and dystrophies, it is of vital importance to correctly interpret these initial trials. Afterall, transplanted cells have no chance of improving vision if they cannot survive the host’s immune reaction.

Investigations in the immunology of solid organ transplants have much to teach us regarding the reaction to RPE grafts. The innate and acquired immune systems cooperate but reject foreign cells by different mechanisms. Studies of RPE transplants have primarily focused on rejection effected by lymphocytes and attempted to prevent this with T-cell inhibitors and steroids. Here we have presented a case based on chronology, histology, and physiology that rejection by microglia and macrophages of the innate immune system should be regarded with increased appreciation. Furthermore, preclinical studies have demonstrated specific interventions that alter the microglial phenotype and should be considered for future trials.

While immune privilege of the eye is not the holy grail it was once hoped to be, understanding the physiology suggests mechanisms to minimize immune recognition. Implantation distant from the retinotomy is most likely to enhance immune separation. Alterations to RPE such as increasing expression of TGFβ or reducing MHC-II will support the effector blockade. An ACAID-type mechanism may allow for priming of host with graft antigens similar to engraftment.

Without available histology, investigators may make educated guesses regarding the health of grafted cells through fundoscopy, OCT, FA, and AF. Expansion of a flat pigmented layer and a healthy appearing neuroretina are good signs, while inflammation, subretinal deposition of material, and disintegration of the transplant all indicate pending or past rejection. However, all of these findings are potentially confounded by physiologic reactions to surgical trauma and inflammation. Measures such as systemic DSA, PBMC reactivity, or GFP fluorescence offer objective and quantifiable evidence of rejection and should be utilized in future investigations.

There are a variety of strategies to prevent immune rejection of transplanted RPE. Immunodeficient hosts are a good option for preclinical research. Xenographic studies of RPE transplants have employed some combination of T-cell inhibitors with steroidal anti-inflammatories. There is evidence that systemic T-cell inhibitors may be more important than systemic steroids for prevention of rejection, but local steroids offer a clear benefit. Allogeneic studies have used systemic tacrolimus and MMF with apparent success as well as intraocular steroids. Studies in basic research suggest that promoting transition of M1 to M2 microglia has potential benefits to both the graft and overlying retina, but this strategy is untested at the level of translational research. For future transplants in clinical patients, matching of banked cells will reduce immunogenicity of the graft. It is likely that researchers and clinicians who employ a combination of these strategies, tailored to their specific graft and host, will have the greatest chance of avoiding immunologic rejection.

## Author Contributions

CP for reviewing the literature in the field and compiling the manuscript. AP for lending his expertise regarding the immune physiology of the retina and potential strategies for evading rejection. VC-S for contributing her expertise in the field of stem cell transplantation. All authors contributed to the article and approved the submitted version.

## Funding

Gates Grubstake Award (GGF012-18-01), Gates Frontiers Fund, The Solich Fund, CellSight Development Fund, and an unrestricted Research Award from Research to Prevent Blindness to the Department of Ophthalmology, University of Colorado.

## Conflict of Interest

The authors declare that the research was conducted in the absence of any commercial or financial relationships that could be construed as a potential conflict of interest.
